# Identification of immune-related gene signature for predicting prognosis in uterine corpus endometrial carcinoma

**DOI:** 10.1038/s41598-023-35655-x

**Published:** 2023-06-07

**Authors:** Siyuan Song, Haoqing Gu, Jingzhan Li, Peipei Yang, Xiafei Qi, Jiatong Liu, Jiayu Zhou, Ye Li, Peng Shu

**Affiliations:** 1 Jiangsu Provincial Hospital of Chinese Medicine, Nanjing, 210029 Jiangsu Province China; 2 Nanjing University of Chinese Medicine, Nanjing, 210029 Jiangsu Province China

**Keywords:** Cervical cancer, Cancer therapy

## Abstract

The objective of this study is to develop a gene signature related to the immune system that can be used to create personalized immunotherapy for Uterine Corpus Endometrial Carcinoma (UCEC). To classify the UCEC samples into different immune clusters, we utilized consensus clustering analysis. Additionally, immune correlation algorithms were employed to investigate the tumor immune microenvironment (TIME) in diverse clusters. To explore the biological function, we conducted GSEA analysis. Next, we developed a Nomogram by integrating a prognostic model with clinical features. Finally, we performed experimental validation in vitro to verify our prognostic risk model. In our study, we classified UCEC patients into three clusters using consensus clustering. We hypothesized that cluster C1 represents the immune inflammation type, cluster C2 represents the immune rejection type, and cluster C3 represents the immune desert type. The hub genes identified in the training cohort were primarily enriched in the MAPK signaling pathway, as well as the PD-L1 expression and PD-1 checkpoint pathway in cancer, all of which are immune-related pathways. Cluster C1 may be a more suitable for immunotherapy. The prognostic risk model showed a strong predictive ability. Our constructed risk model demonstrated a high level of accuracy in predicting the prognosis of UCEC, while also effectively reflecting the state of TIME.

## Introduction

Uterine Corpus Endometrial Carcinoma (UCEC) is a malignant tumor of the epithelium of the endometrial tissue, which can infiltrate the myometrium as the disease progresses, accounting for 20–30% of malignant tumors of the female genital tract. It poses a serious threat to women's health^1^. UCEC is easily curable when diagnosed early. However, metastatic UCEC is a common disease^2^. At present, it is generally believed that it is related to the hyperstimulation of the uterus by estrogen^3^. Treatment is mostly surgical or oral medications that regulate estrogen and progesterone, but surgery is costly and oral medications predispose to recurrence^4^.

Studies have shown that the tumor immune microenvironment (TIME) plays an important role in the occurrence and development of tumor cells and is closely related to tumor prognosis recently^5,6^. At present, it is generally believed that the existence of an immune microenvironment can inhibit tumor growth and prevent tumor metastasis^7^. UCEC genesis is essentially a chronic inflammatory process involving immune cells^8^. Cytokines secreted by immune infiltrating cells have a certain impact on the survival of UCEC patients^1,9^. For example, the production of TAMs is related to circulating monocytes. The higher the number of circulating monocytes in patients, the higher the incidence of pelvic lymph node metastasis and myometrial invasion^10^. The level of Treg cells in UCEC patients is significantly increased, indicating that Treg cells can inhibit the infiltration and metastasis of tumor cells^11^.

Bioinformatics is an approach to mine and analyze target data based on public databases, thereby providing useful evidence for disease biomarker discovery and targeted drug research^12,13^. Based on TCGA and GEO expression profiling data, this study clustered UCEC according to molecular-specific subgroups associated with ICI patterns, and identified reliable diagnostic and prognostic biomarkers.

## Method

### Data download and processing

As a training cohort, we downloaded data sets and clinical information for UCEC from the TCGA database (https://portal.gdc.cancer.gov/). The mRNA expression dataset from GSE17025 based on platform GPL570 was selected and acquired from the GEO database (https://www.ncbi.nlm.nih.gov/geo) as a validation dataset. The clinical features were shown in Supplementary Table S1.

### Overview of TIME in UCEC

To assess the TIME in UCEC, we used the ESTIMATE package to evaluate immune cell subsets and immune signatures, the higher the corresponding score, the higher the proportion of the corresponding component in TIME^14^. The UCEC samples were clustered into discrete subgroups using the ConsesusClusterPlus package^15^. Using agglomerative Pam clustering with a *1-pearson* correlation distance and 80% of the samples were sampled 10 times. After that, we conducted survival analysis by Kaplan–Meier curves (http://kmplot.com/) in each cluster.

### Correlation of immune cell infiltration

To evaluate the correlation of immune cells during infiltration, the TIME was characterized by CIBERSORT^16^ to compare the relative subpopulations of immune cells and immune scores among the three different immune clusters, and *P* < 0.05 were considered eligible for subsequent analysis. Immune infiltration correlation matrices were constructed with absolute values of weak correlation coefficients between 0.10 and 0.39, moderate correlation coefficients between 0.40 and 0.69, and strong correlation coefficients between 0.7 and 0.89^17^. In addition, to further evaluate the prognostic value of different immune infiltrating cells, survival analysis was performed according to different immune cell subsets.

### Correlation between ICI score and immunotherapy

Immunotherapy with immune checkpoint inhibitors (ICI) has dramatically changed cancer treatment strategies, therefore, we analyzed the correlation of ICI score with the expression levels of six key genes related to immune checkpoint blockade (CTLA-4, PD-1, PD-L1, PD-L2, TIM-3, and LAG3)^18^.

### Functional enrichment analysis in the training cohort

The DEGs between cluster C1, C2, and C3 in the training cohort were analyzed with the Limma package^19^. Set threshold *P* < 0.05 and |logFC| ≥ 1. To further elucidate the biological roles of DEGs, the DAVID website (https://david.ncifcrf.gov/) was used for functional enrichment analysis^20^. Bubble charts were created using R language. The PPI network was constructed through the STRING website (https://cn.string-db.org/) and visualized with Cytoscape 3.7.2.

### Identification of hub genes in different database

The tumor mutational burden (TMB) of hub genes was calculated by Maftools, using the ggplot2 package^21^ to draw the distribution map of mutation. We used GEPIA (http://gepia.cancer-pku.cn/index.html) to verify the expression levels of hub genes in tumor and normal tissues as well as the survival analysis^22^. The correlation between hub genes and the infiltration level of immune cells (B cells, CD4 + T cells, CD8 + T cells, neutrophils, macrophages, and dendritic cells) was investigated on Tumor Immune Estimation Resource (TIMER) website (https://cistrome.shinyapps.io/timer/)^23^.

### WGCNA and identification of key module in the validation cohort

The WGCNA package^24^ was used to construct a co-expression network. The method mainly consisted of the following steps: first, a weight coefficient β was selected. Second, the gene expression profiles were transformed into adjacency matrices, which were used to define separate similarity measures. Finally, module eigengenes were calculated for each module. We selected the hub genes in the black module significantly related to the grade for GSEA (version 1.52.1) analysis. Terms with FDR < 0.05 were visualized by the ggplot2 R package to investigate the potential functions of the hub genes^25^.

### Construction and verification of prognostic risk signature

To identify prognostic genes, a univariate Cox regression analysis of OS was performed. The risk signature was constructed using LASSO analysis combined with clinical information, and the samples were divided into high-risk groups and low-risk groups by Kaplan–Meier curves^26^. The expression levels, survival analysis, and the immune cells infiltration levels of prognostic genes were verified by GEPIA and TIMER. The infiltration level for each SCNA category is compared with the normal using a two-sided Wilcoxon rank-sum test. The “RMS” R package was used to draw a nomogram to predict the possibility of OS. A ROC curve was constructed using the survival ROC R package to predict the prognosis of the model in the training cohorts. The CIBERSORT package was used to describe the TIME characteristic between the two groups. The GSE17025 data set was used to verify the accuracy of the prognosis model.

### Experimental verification in vitro

#### Cell culture

Endometrial epithelial cells (EECs) was purchased from Wuhan Procell Life Science&Technology Co.,Ltd (catalog number: CM-H058), and endometrial cancer cells HEC-1A was purchased from Nanjing KGI Biological Company (catalog number: KG626). They were cultured in DMEM containing 10% fetal bovine serum, 100 U/ml penicillin, and streptomycin in a box at a constant temperature of 37 °C (where the CO_2_ concentration was 5%), and passaged when the cell density increased to 50–70%.

#### Quantitative real‑time PCR

According to the method of Trizol to extract total RNA in cells and detect the integrity and purity of the total RNA, the reverse transcription reagent box will RNA into cDNA, and then use fluorescence quantitative PCR instrument for fluorescence quantitative detection. The PCR cycle program consisted of 35 cycles of preheating at 95 °C for 3 min, followed by denaturation at 94 °C for 20 s, annealing at 59 °C for 30 s, and extension at 72 °C for 20 s. The 2^−ΔΔCt^ method was used to calculate the relative expression level. The specific sequences of the primers are shown in Supplementary Table S2.

### Statistical analysis

The data in this study were analyzed using R (3.6.1). The *t*-test was used to measure differences between two groups, and the comparison between multiple groups used the Kruskal–Wallis test. “glmnet” package was used for LASSO analysis. For other tests, a *p* < 0.05 was considered statistically significant.

## Results

### TIME landscape of UCEC

**Figure 1:**
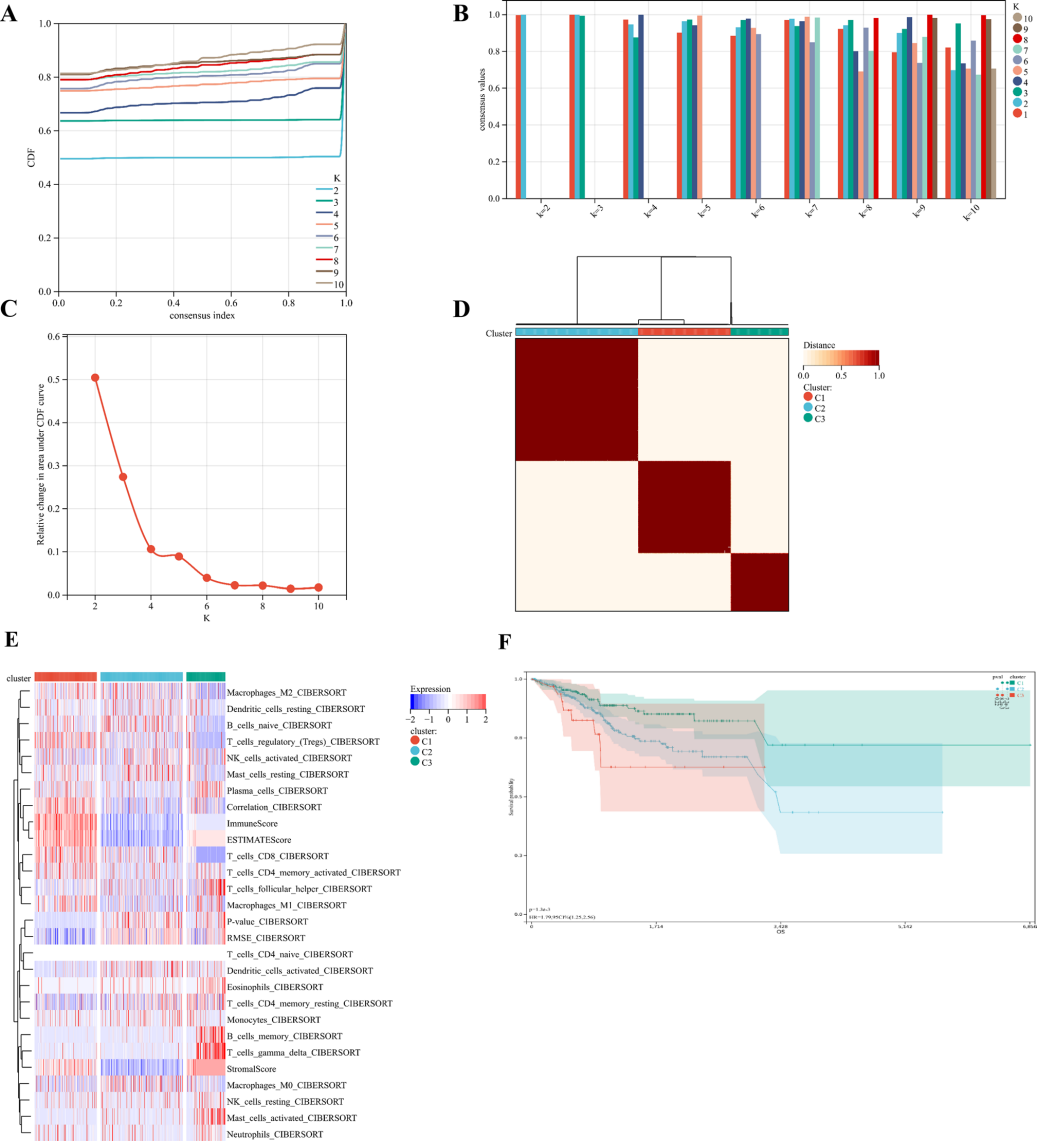

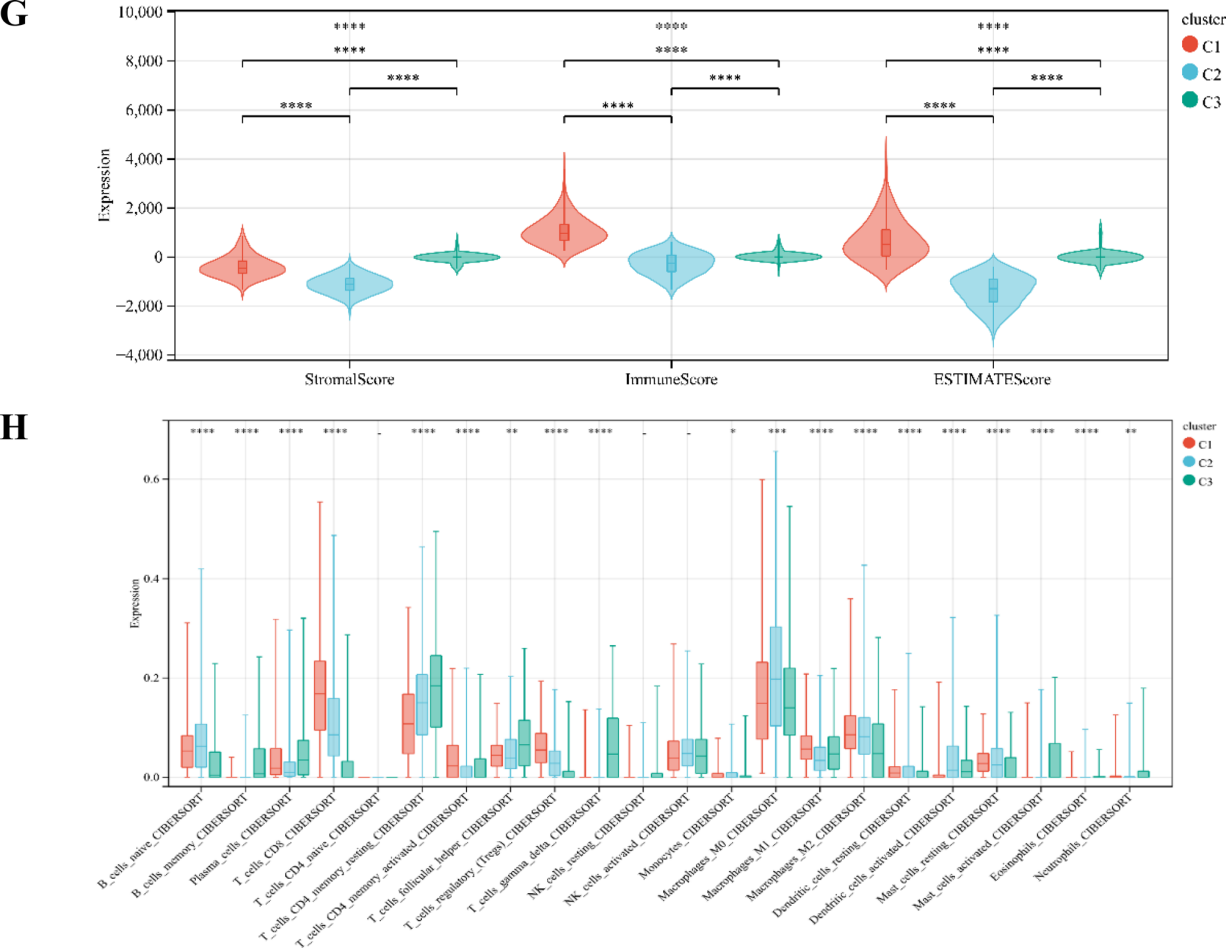
Tumor immune microenvironment landscape of UCEC. (**A**–**D**) Sample clustering consistency CDF curve, histogram, line graph, and heatmap. (**E**) An unsupervised cluster heatmap of immune cell infiltration in patients with UCEC. Rows represent immune infiltrated cells and Columns represent samples. (**F**) Kaplan–Meier survival curves of three clusters. (**G**) Stromal Score, Immune Score, and ESTIMATE Score in three clusters (*p < 0.05; **p < 0.01; ***p < 0.001). (**H**) Subsets of immune cell infiltration in three clusters (*p < 0.05; **p < 0.01; ***p < 0.001).

Three immune infiltration clusters were identified by using "ConsesusClusterPlus", including cluster C1 (219 samples), cluster C2 (290 samples), and cluster C3 (137 samples) (Fig. 1A–D). To further reveal the potential relationship between ICI and immune infiltrating cells, the integrated landscape of TIME was visualized as a heatmap (Fig. 1E). Kaplan Meier survival analysis showed that the median survival advantage was evident in the cluster C1, whereas the cluster C3 had the worst prognosis (Fig. 1F). Cluster C1 showed the highest Stroma Score, Immune Score and ESTIMATE Score, while cluster C2 showed the lowest Stroma Score, Immune Score and ESTIMATE Score (Fig. 1G). Cluster C1 was characterized by a marked increase in antitumor cell subsets such as CD8 + T lymphocytes, activated memory CD4 + T lymphocytes, and M1 and M2 macrophages. Cluster C2 was characterized by a marked increase in inflammatory cells such as naive B lymphocytes, M0 macrophages, and mast cells. Cluster C3 was characterized by a marked increase in plasma cells, memory CD4 + T lymphocytes, and helper T lymphocytes (Fig. 1H). Accordingly, we found that cluster C1 had the highest immune score, whereas cluster C2 had the lowest immune score. The TIME is usually divided into three categories: immune inflammation, immune rejection, and immune desert^27^. These results demonstrated that the TIME and immune status of the three clusters differed significantly. Cluster C1 with a good prognosis had a high immune status, so we speculate that cluster C1 represents the immune inflammation type, cluster C2 represents the immune rejection type, and cluster C3 represents the immune desert type.

### Correlation of immune cell infiltration

**Figure 2:**
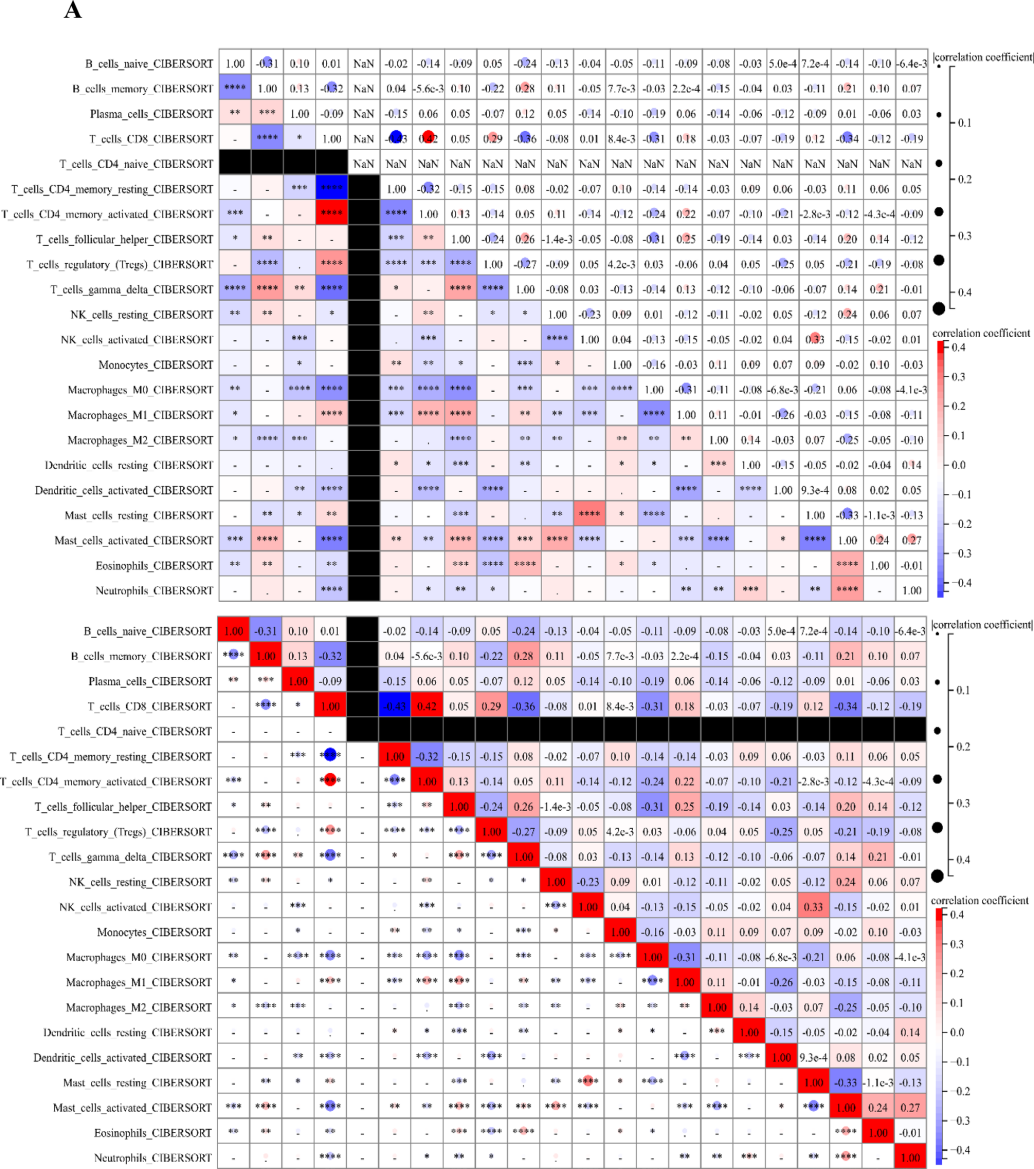

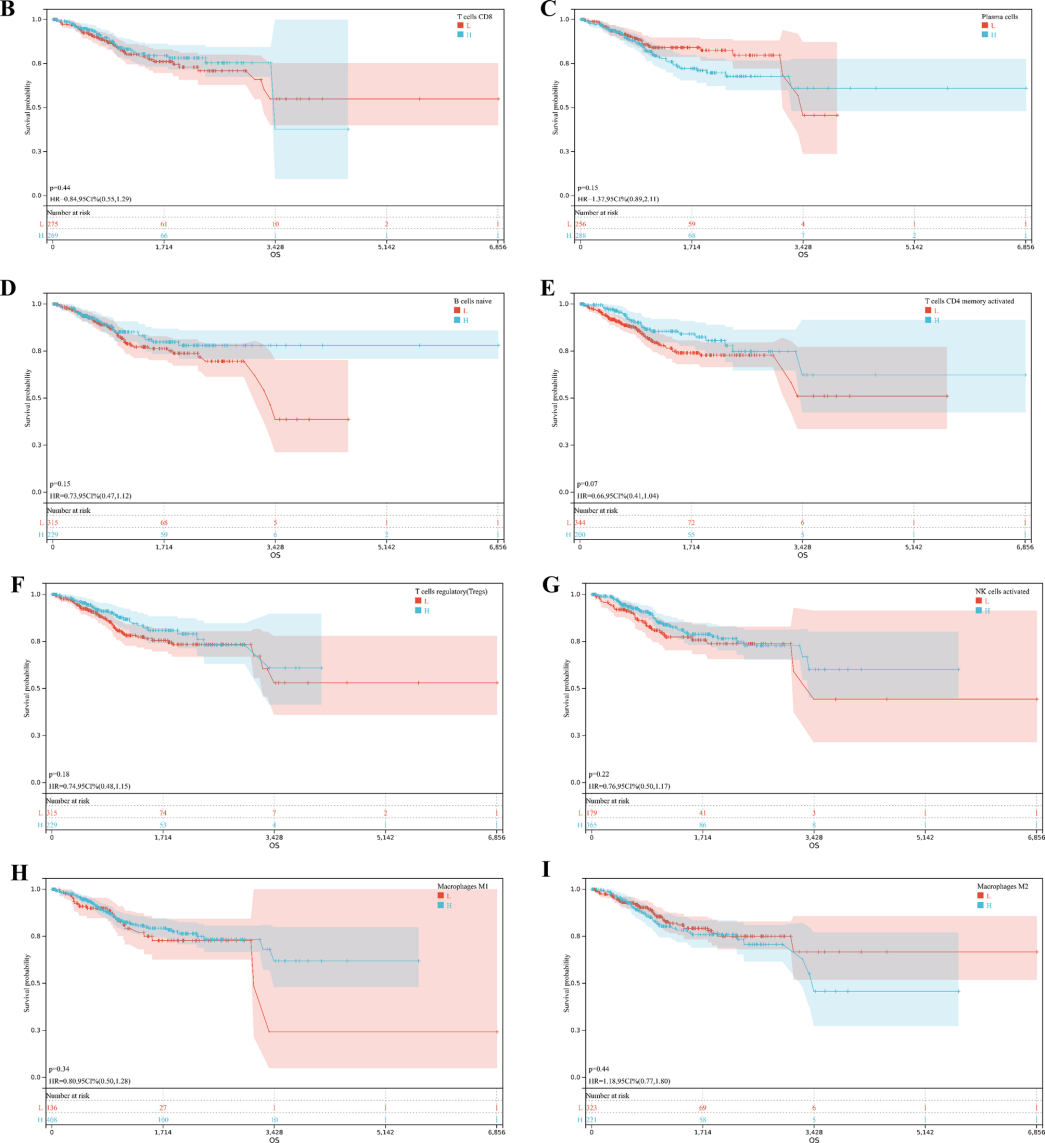
Immune cell infiltration patterns with different immunophenotypic features. (**A**) Immune infiltration correlation matrix. The fraction of immune cells was positively related and represented in red, whereas others were negatively related and represented in blue. p < 0.05 was the cut-off. (**B**) Analysis of survival probability among patients with different T cells CD8 infiltration levels. (**C**) Analysis of survival probability among patients with different Plasma infiltration levels. (**D**) Analysis of survival probability among patients with different B cells naive infiltration levels. (**E**) Analysis of survival probability among patients with different T cells CD4 memory activated infiltration levels. (**F**) Analysis of survival probability among patients with different T cells regulatory (Tregs) infiltration levels. (**G**) Analysis of survival probability among patients with different NK cells activated infiltration levels. (**H**) Analysis of survival probability among patients with different Macrophages M1 infiltration levels. (**I**) Analysis of survival probability among patients with different Macrophages M2 infiltration levels.

We characterized TIME with CIBERSORT to compare the correlation of immune cell infiltration in three different clusters (Fig. 2A), showing that CD8 + T lymphocytes were moderately correlated with activated memory CD4 + T lymphocytes, activated NK cells were weakly correlated with mast cells, and CD8 + T lymphocytes were weakly correlated with Treg cells. Survival analysis showed that activated CD4 memory cells, naive B cells, M1 macrophages, Treg cells, and activated NK cells infiltrating were found to be associated with longer survival, while plasma cell and M2 macrophage infiltration had a worse prognosis (Fig. 2B–I).

**Figure 3:**
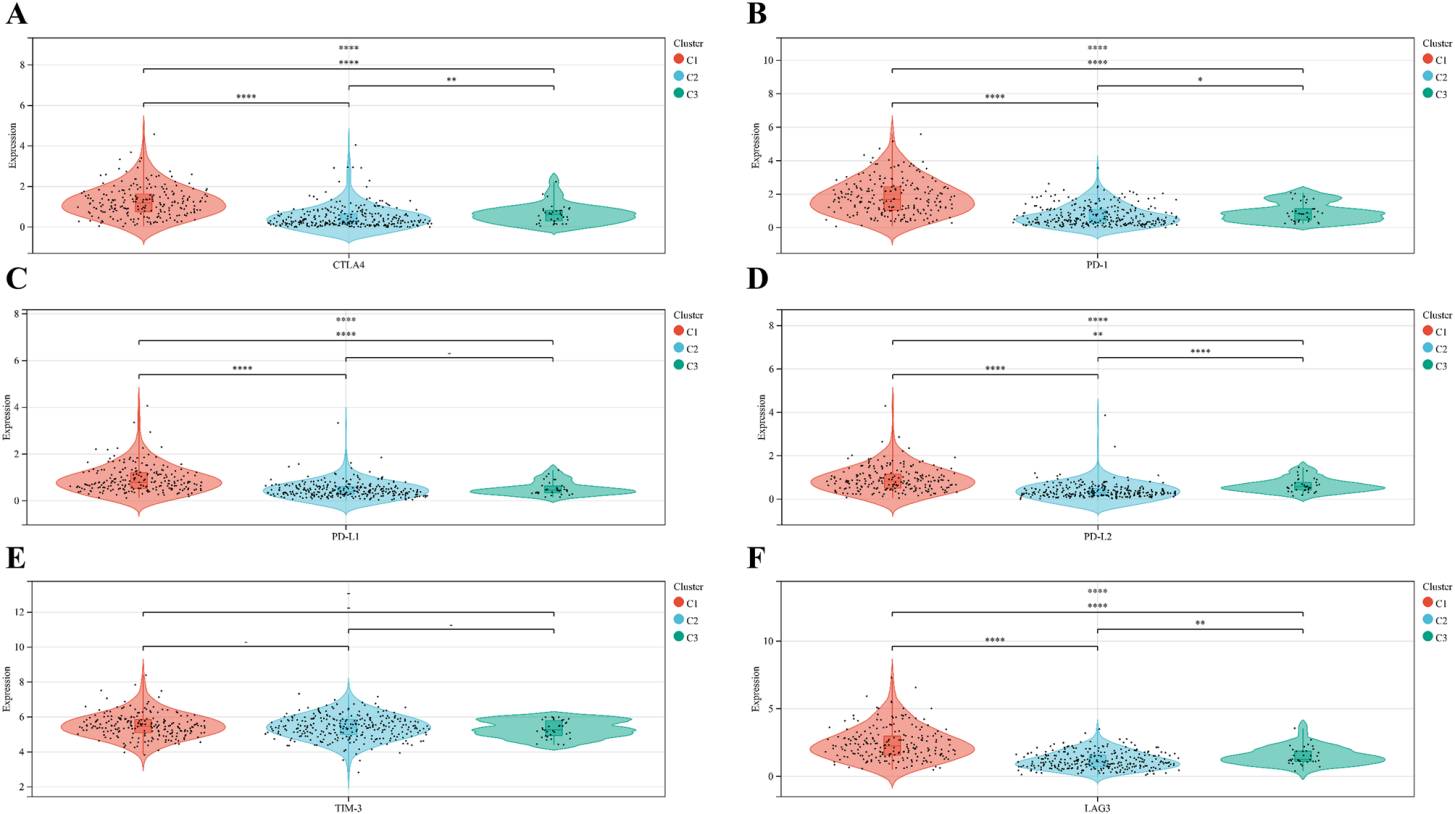
Expression levels of immune checkpoint-associated genes in different ICI clusters of UCEC. (**A**) CTLA4 expression levels in different clusters. (**B**) PD-1 expression levels in different clusters. (**C**) PD-L1 expression levels in different clusters. (**D**) PD-L2 expression levels in different clusters. (**E**) TIM-3 expression levels in different clusters. (**F**) LAG3 expression levels in different clusters. (*p < 0.05; **p < 0.01; ***p < 0.001).

We compared the expression levels of six key immune checkpoint repressor genes in the three clusters. The results showed that cluster C1 was characterized by the highest expression levels of immune checkpoint repressor genes, while cluster C3 with the lowest expression levels of immune checkpoint repressor genes (Fig. 3A–F), suggesting that cluster C1 may be more suitable for immunotherapy.

### Functional enrichment analysis in the training cohort

**Figure 4:**
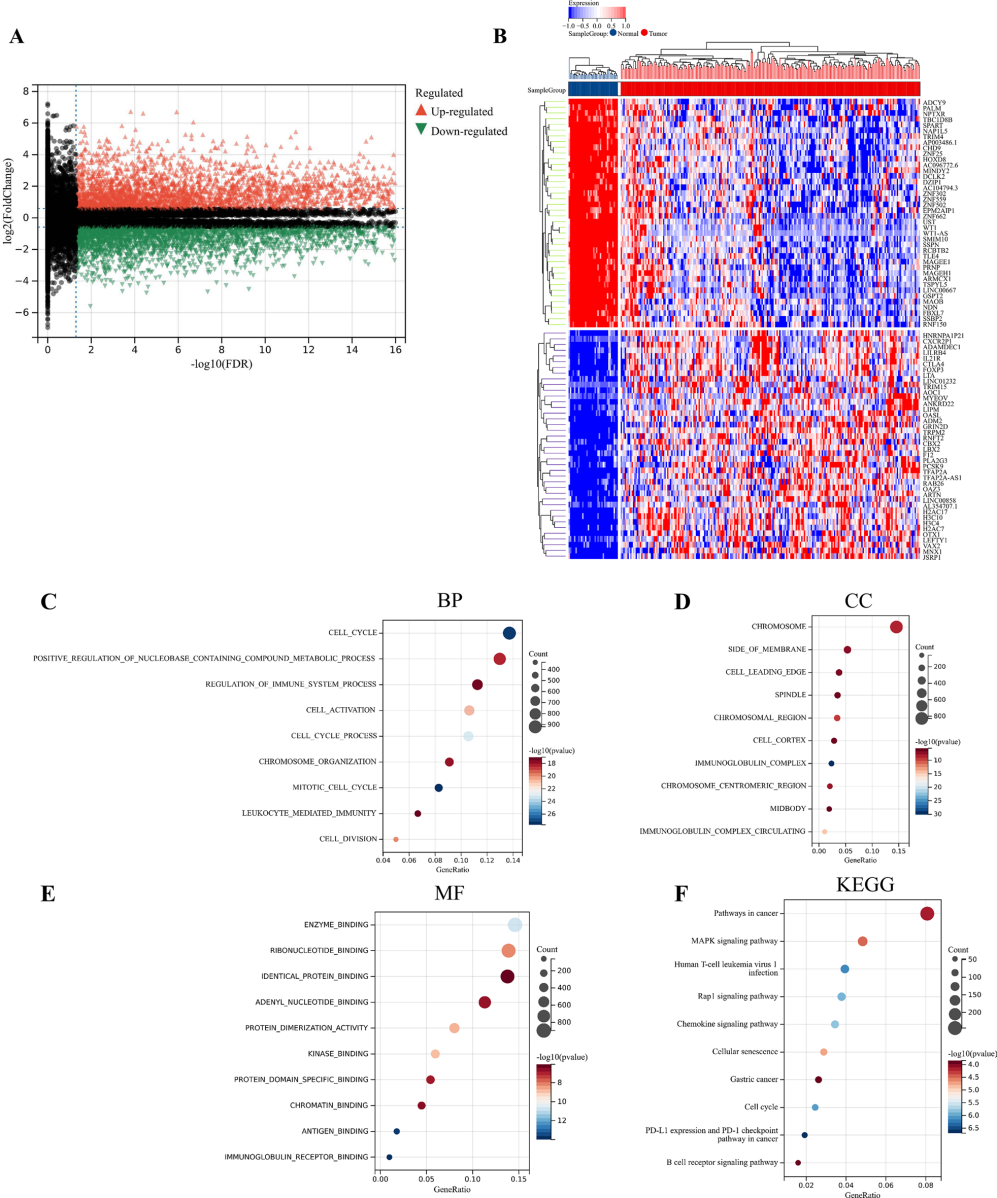
Identification and enrichment analysis of DEGs in training cohort. (**A**) Volcanic map of DEGs. (**B**) Heatmap of DEGs. (**C**) Biological Process of DEGs. (**D**) Cellular Components of DEGs. (**E**) Molecular Function of DEGs. (**F**) KEGG^59^ of DEGs.

**Figure 5:**
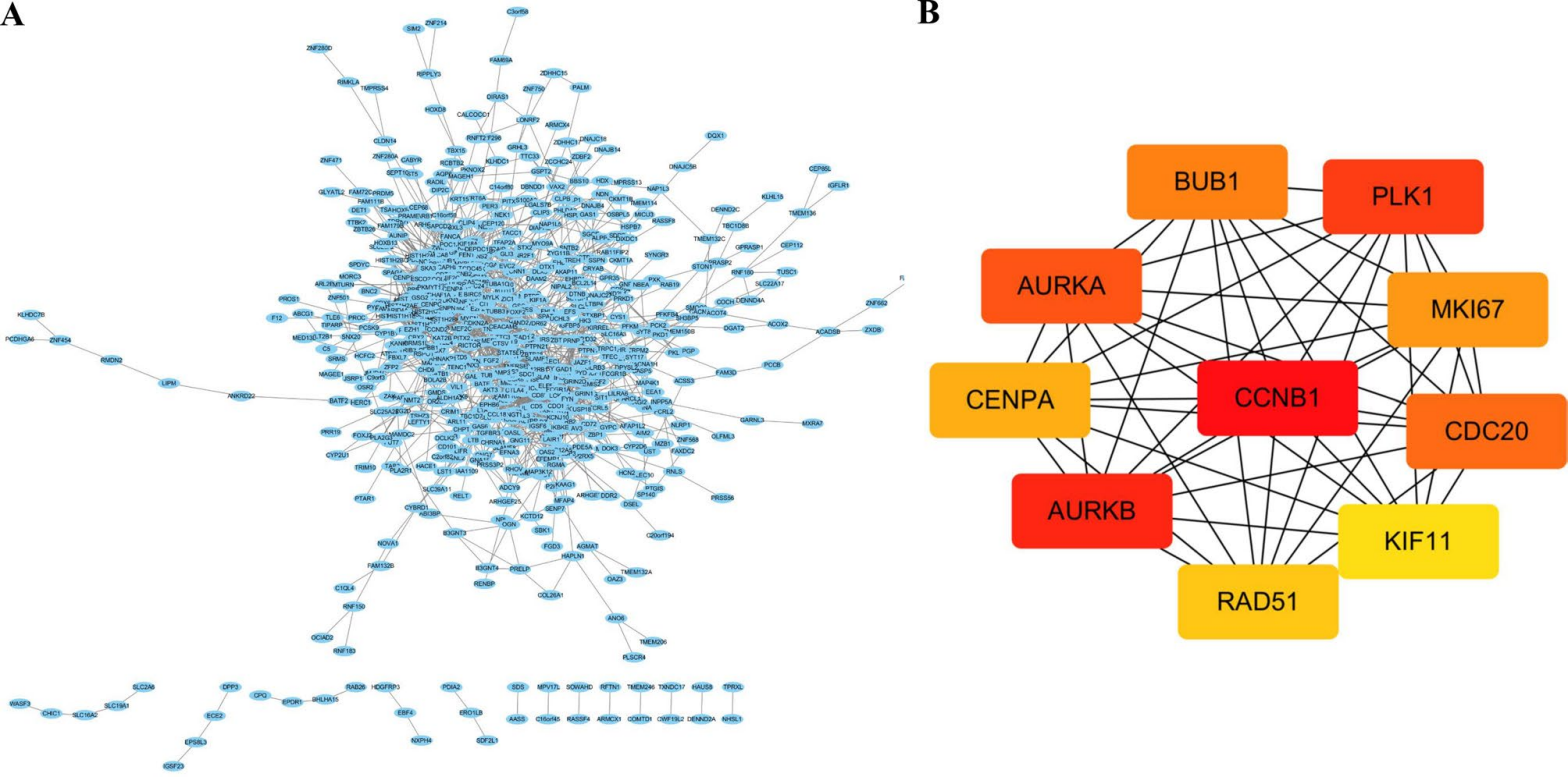
PPI network and hub genes based on DEGs in the training cohort. (**A**) PPI network based on DEGs. (**B**) Hub genes based on PPI network.

A total of 5571 DEGs were identified using the “Limma” R package, including 2713 up-regulated genes and 2858 down-regulated genes (Fig. 4A,B). GO enrichment analysis showed that biological processes mainly involved cell cycle, cell activation, and cell cycle process. Cell components mainly include the chromosome, side of the membrane, and spindle. Molecular functions mainly include enzyme binding, ribonucleotide binding, and identical protein binding. KEGG enrichment analysis mainly involved the MAPK signaling pathway, PD-L1 expression, and PD-1 checkpoint pathway in cancer. The top ten bubbles were plotted in Fig. 4C–F. Moreover, the entire PPI network was analyzed using cytoHubba (Fig. 5A), the top 10 genes were screened out by taking p value as a standard, including BUB1, PLK1, MKI67, CDC20, KIF11, RAD51, AURKB, CENPA, AURKA, and CCNB1 (Fig. 5B). These hub genes were considered as risk factors for UCEC.

### Identification of hub genes in different database

**Figure 6:**
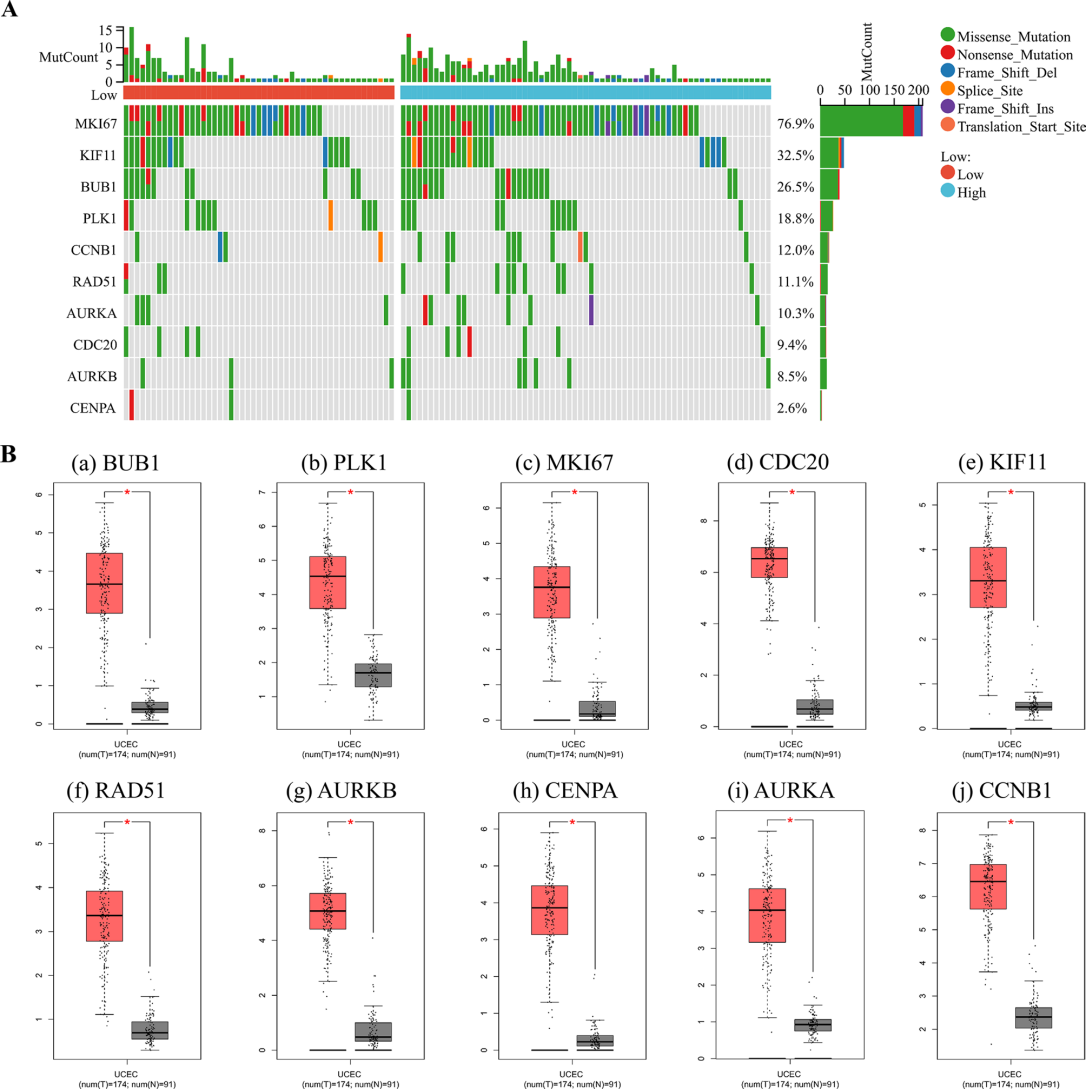

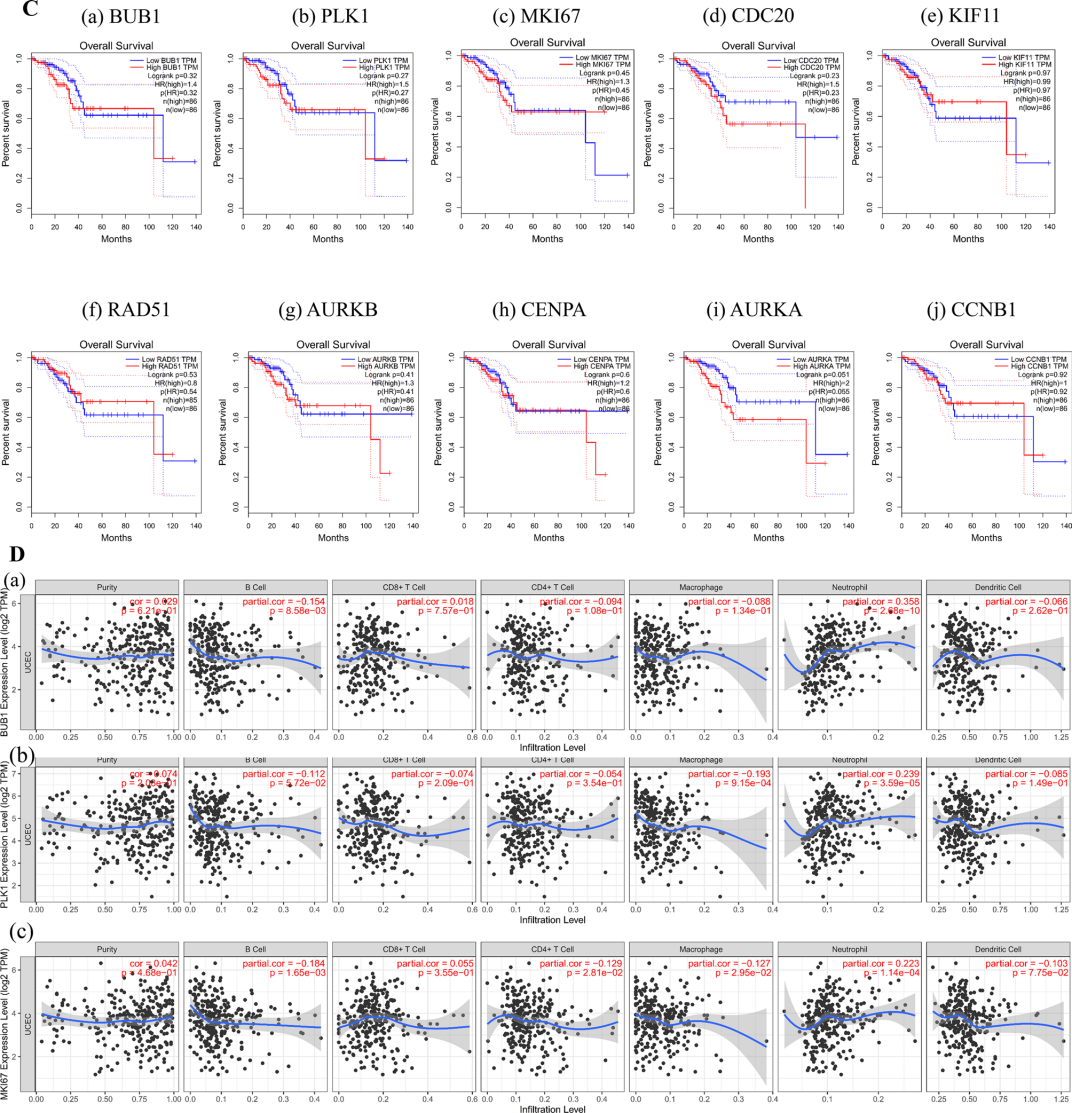

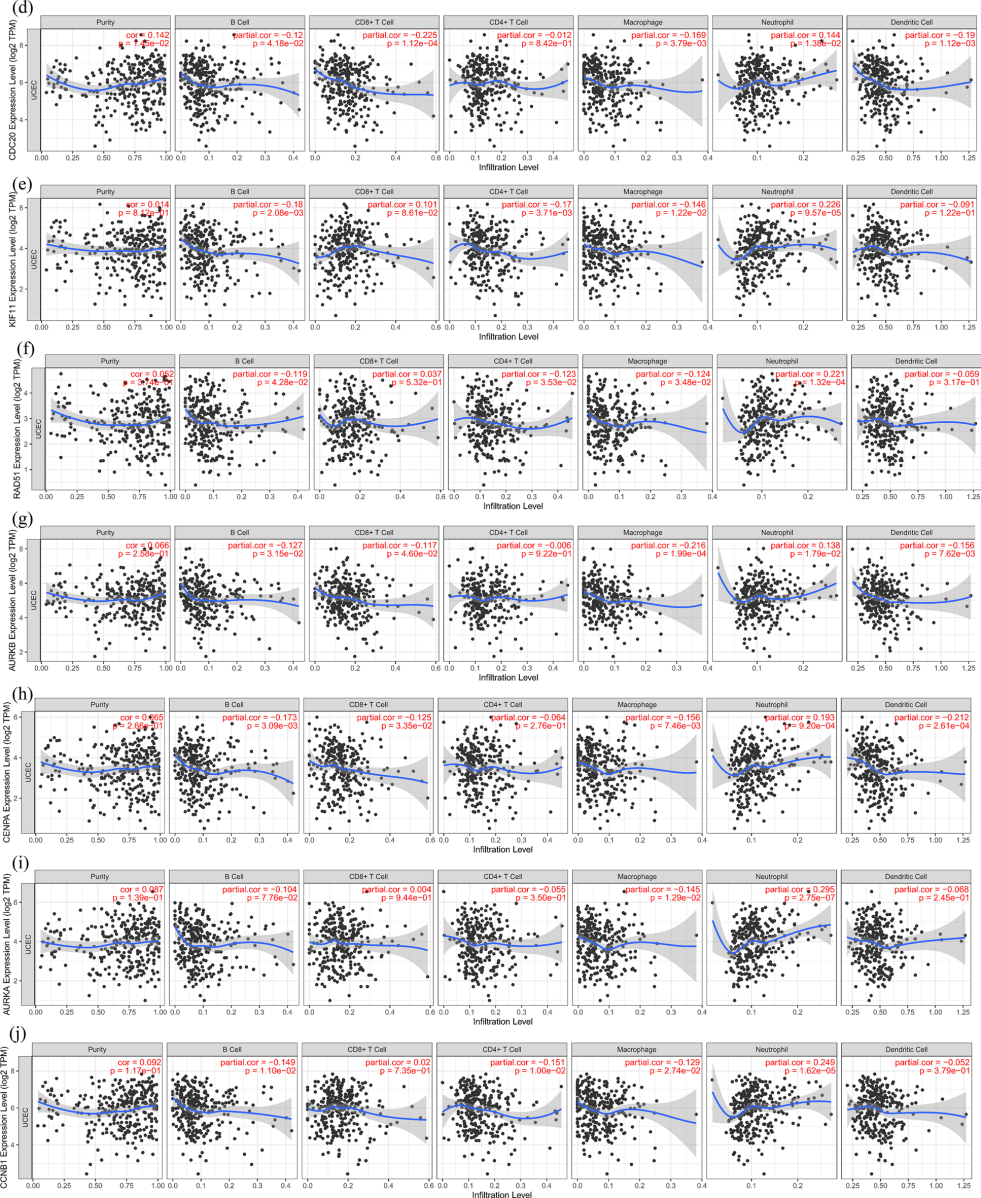
Identification of hub genes in different database. (**A**) Correlation of hub genes with tumor mutational burden. (**B**) Expression of hub genes (a) BUB1, (b) PLK1, (c) MKI67, (d) CDC20, (e) KIF11, (f) RAD51, (g) AURKB, (h) CENPA, (i) AURKA, (j) CCNB1 in GEPIA. Red represents tumor, and gray represents normal. (**C**) Overall survival analysis hub genes (a) BUB1, (b) PLK1, (c) MKI67, (d) CDC20, (e) KIF11, (f) RAD51, (g) AURKB, (h) CENPA, (i) AURKA, (j) CCNB1 in GEPIA. (**D**) Correlation between the a) BUB1, (b) PLK1, (c) MKI67, (d) CDC20, (e) KIF11, (f) RAD51, (g) AURKB, (h) CENPA, (i) AURKA, (j) CCNB1 and the infiltration level of immune cells in TIMER database.

We observed the highest mutation rate of 76.9% for MKI67, with other hub genes mutated to varying degrees in UCEC (Fig. 6A). GEPIA showed that hub genes were all expressed highly in UCEC, and they differed significantly between normal and tumor (Fig. 6B). Except overall survival of AURKA expression in UCEC was significantly different, other hub genes had no significant difference (Fig. 6C). The TIMER noted a positive relationship between CDB + T cells and expression of BUB1 (Cor = 0.018, p = 7.57e−01), MKI67 (Cor = 0.055, p = 3.55e−01), KIF11 (Cor = 0.101, p = 8.61e−02), RAD51 (Cor = 0.037, p = 5.32e−01), AURKA (Cor = 0.004, p = 9.44e−01), and CCNB1 (Cor = 0.02, p = 7.35e−01). Neutrophils was positively correlated with expression of BUB1 (Cor = 0.358, p = 2.68e−10), PLK1 (Cor = 0.239, p = 3.59e−05), MKI67 (Cor = 0.223, p = 1.14e−04), CDC20 (Cor = 0.144, p = 1.38e−02), KIF11 (Cor = 0.226, p = 9.57e−05), RAD51 (Cor = 0.221, p = 1.32e−04), AURKB (Cor = 0.138, p = 1.79e−02), CENPA (Cor = 0.193, p = 9.20e−04), AURKA (Cor = 0.295, p = 2.75e−07), and CCNB1 (Cor = 0.249, p = 1.62e−05) (Fig. 6D).

### WGCNA and identification of key module in the validation cohort

**Figure 7:**
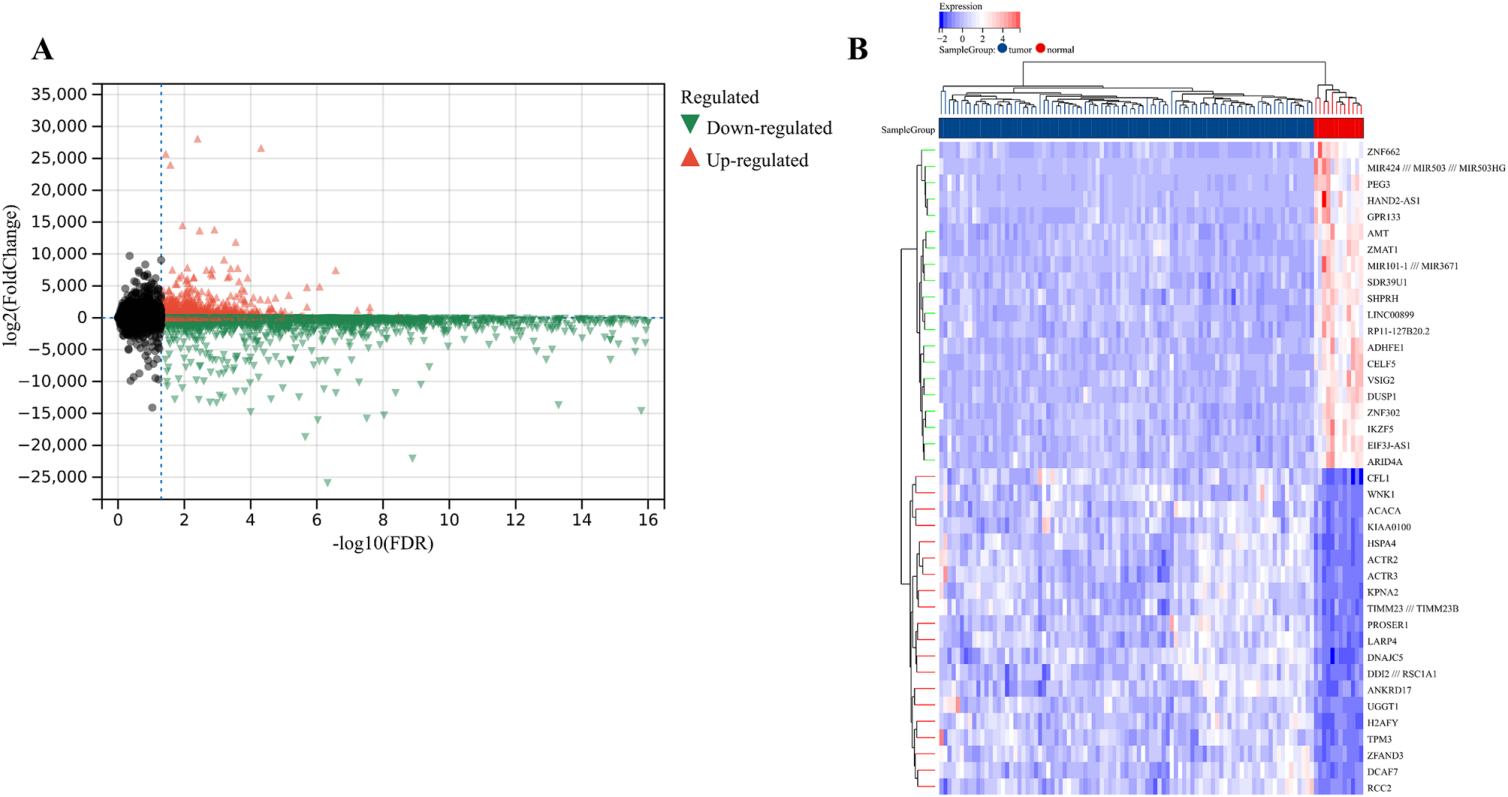
DEGs in the validation cohort. (**A**) Volcanic map of DEGs in the validation cohort. (**B**) Heatmap of DEGs in the validation cohort.

**Figure 8:**
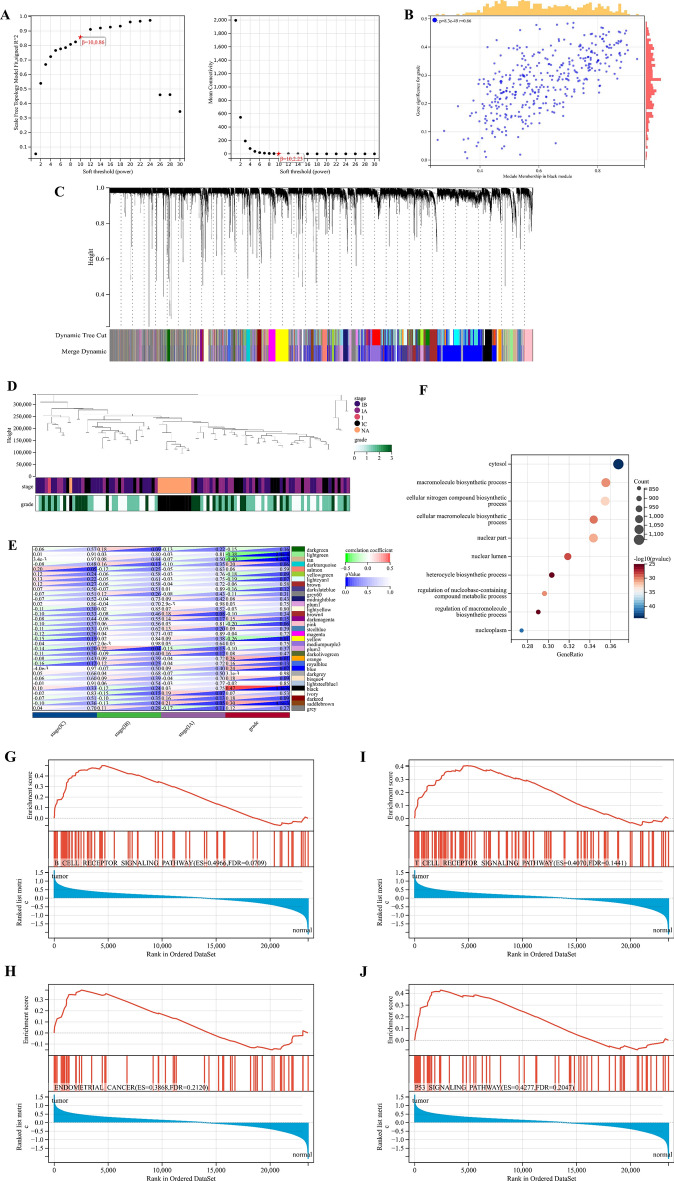
Weighted correlation network analysis (WGCNA) in the validation cohort. (**A**) Analysis of the scale-free fit index (left) and the mean connectivity (right) for various soft-thresholding powers. (**B**) GS and MM correlation scatter. (**C**) Gene clustering dendrograms. (**D**) Heatmap of correlations between sample clusters and clinical features. (**E**) Heatmap of correlations between modules and clinical features. (**F**) GO enrichment analysis of hub genes in the validation cohort. (**G**–**J**) GSEA analysis of hub genes in the validation cohort.

A 1446 up-regulated genes and 2865 down-regulated genes in the validation cohort were screened out (Fig. 7A,B). The up-regulated and down-regulated DEGs were analyzed by WGCNA. We combined the modules with the distance less than 0.25, and finally obtained 33 co-expression modules (Fig. 8A–C). The correlation between sample clusters and clinical features is shown in Fig. 8D,E, and the black was considered as the most significant module. GO enrichment analysis of hub genes is shown in Fig. 8F. We selected the hub genes for GSEA analysis. The B cell receptor signaling pathway, T cell receptor signaling pathway, Endometrial Cancer, and P53 signaling pathway were significantly enriched in the in the black module (Fig. 8G–I).

### Establishment of prognostic risk signature

**Figure 9:**
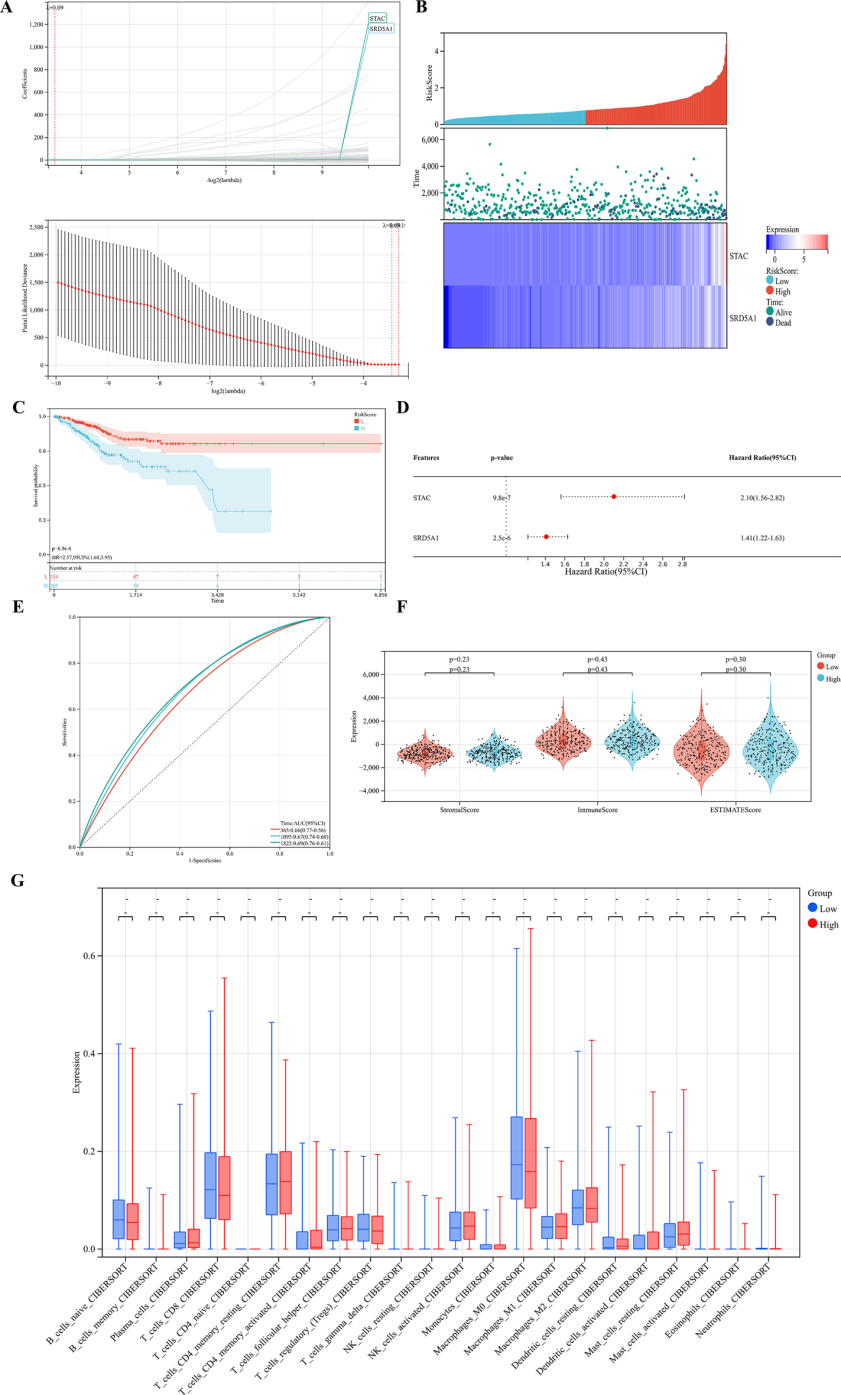
Establishment of prognostic risk signature in the training cohort. (**A**) LASSO analysis with suitable lambda. (**B**) Distribution of survival status, risk score, and heatmap of UCEC patients in the high and low-risk groups. (**C**) Survival curve of the UCEC patients in the two groups. (**D**) Forest map of multi-factor survival analysis. (**E**) Time-dependent ROC curve of the risk signature. (**F**) Stromal score, Immune score, and ESTIMATE score in the high and low-risk groups. (**G**) Statistical analysis of immune-related cells evaluated by CIBERSORT algorithm in the two groups.

**Figure 10:**
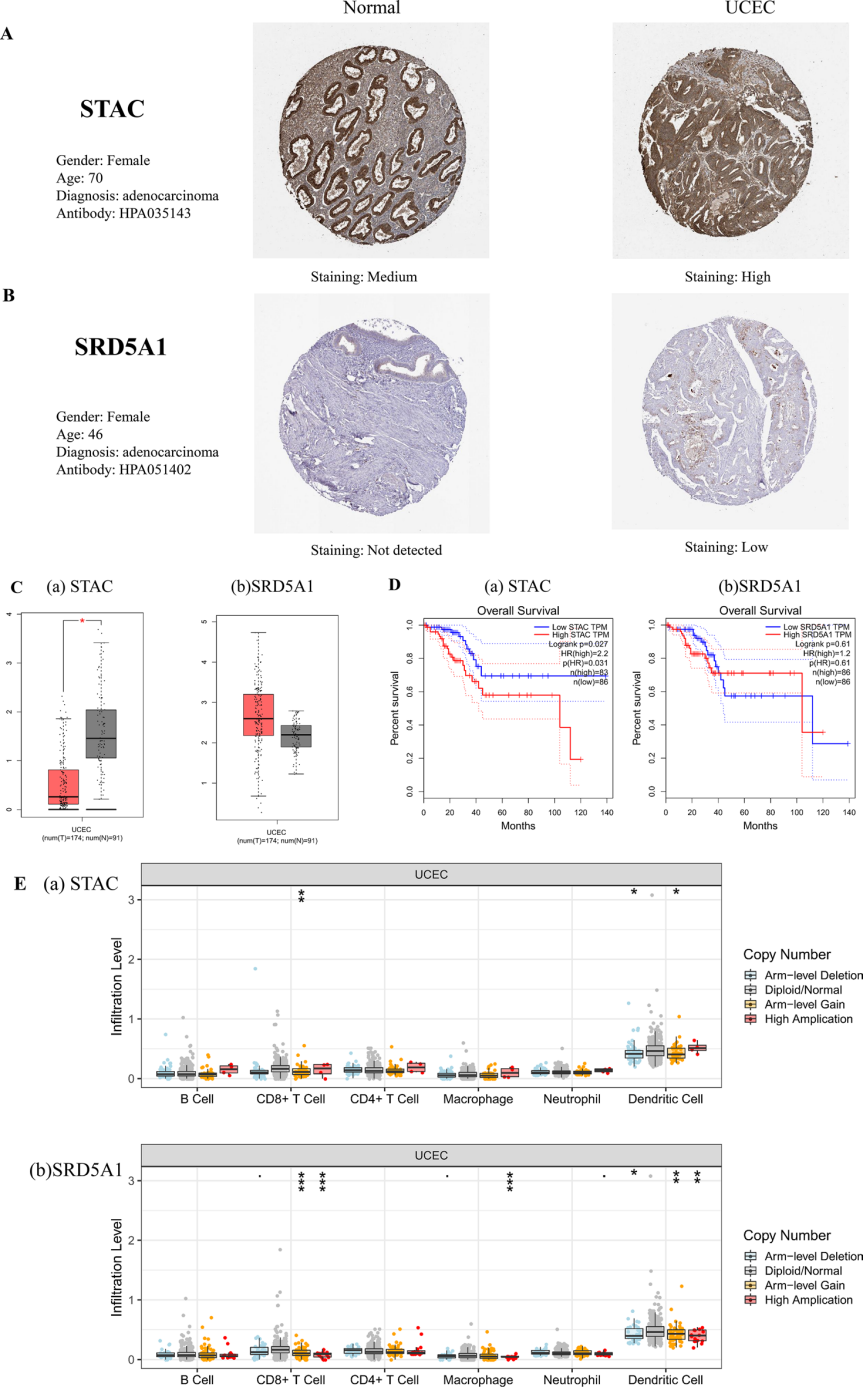
Validation of prognosis genes in different databases. (**A**) The IHC of STAC in the HPA database. (**B**) The IHC of SRD5A in the HPA database. (**C**) Expression of prognosis genes (a) STAC, and (b) SRD5A in GEPIA. Red represents tumor, and gray represents normal. (**D**) Overall survival analysis of prognosis genes (a) STAC, and (b) SRD5A in GEPIA. (**E**) Box plots of tumor infiltration levels with different somatic copy number alterations for prognosis genes.

**Figure 11:**
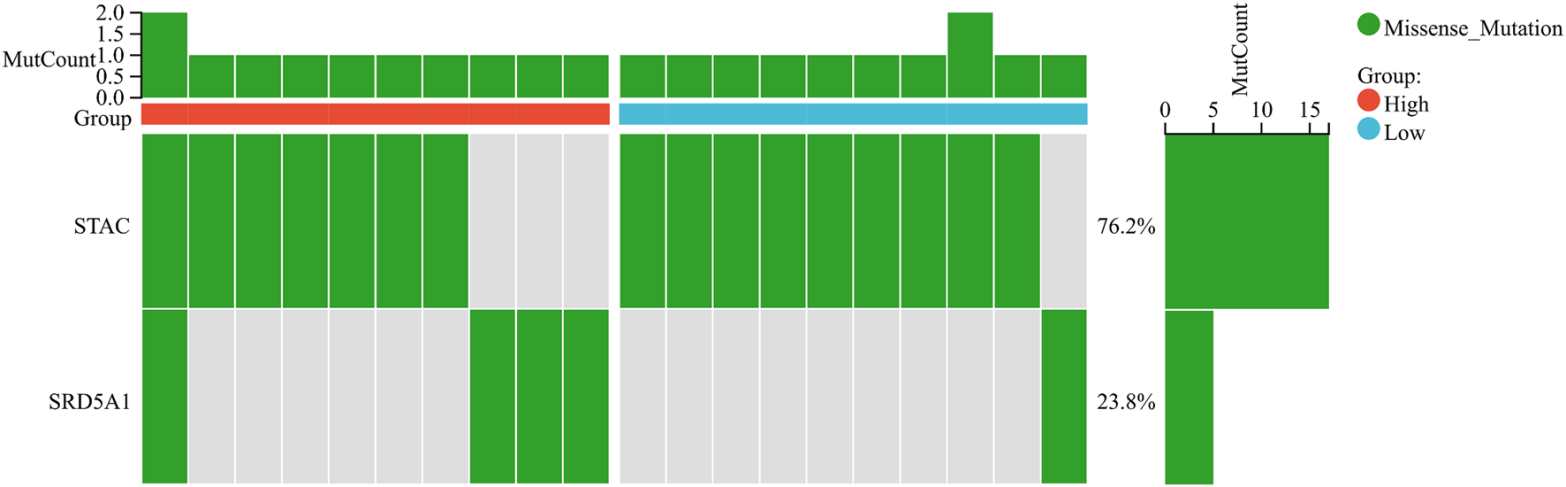
Tumor mutational burden (TMB) of prognosis-related genes.

We signature the risk based on LASSO analysis, tenfold cross-validation was set up to obtain the optimal model. RiskScore = [0.0554916674282798 × SRD5A1 + 0.00263378960689375 × STAC], two prognostic related genes were finally obtained (Fig. 9A). The established risk signature successfully classified the UCEC patients into high-risk and low-risk groups (Fig. 9B). Survival outcomes were significantly lower in patients with high-risk scores than in patients with low-risk scores (Fig. 9C). The Hazard Ratio (HR) of two prognostic genes were greater than 1 (Fig. 9D). In addition, the AUC of risk score of OS in 1 year, 3 years, and 5 years is 0.66, 0.67 and 0.67, respectively (Fig. 9E). Finally, the TIME of the two groups showed that the high-risk group had a higher ESTIMATE score, higher Immune score, and higher Stromal score (Fig. 9F). The CIBERSORT algorithm indicated that CD8 + T cells, activated CD4 memory cells, Tregs, Macrophases-M0, Macrophases-M2, and resting Mast cells were significantly higher in high-risk groups than in low-risk groups, while memory B cells, resting CD4 memory T cells, activated NK cells, Neutrophils, and Macrophases-M0 were significantly higher in low-risk groups than in high-risk groups (Fig. 9G), suggesting the TIME and immune status of the two groups differed significantly. The protein expressions of STAC and SRD5A1 in UCEC and normal tissues were significantly different (Fig. 10A,B). GEPIA showed that the prognostic gene SRD5A1 was expressed highly in UCEC, while the prognostic gene STAC was expressed lowly in UCEC (Fig. 10C). Patients with highly expressed STAC have a short overall survival (*P* < 0.05) (Fig. 10D). STAC and SRD5A1 both had highly CDB + T cells infiltration level, suggesting that the prognostic genes are closely related to TIME (Fig. 10E). In addition, we observed mutations of STAC is 76.2% (Fig. 11).

### Verification of prognostic risk signature

**Figure 12:**
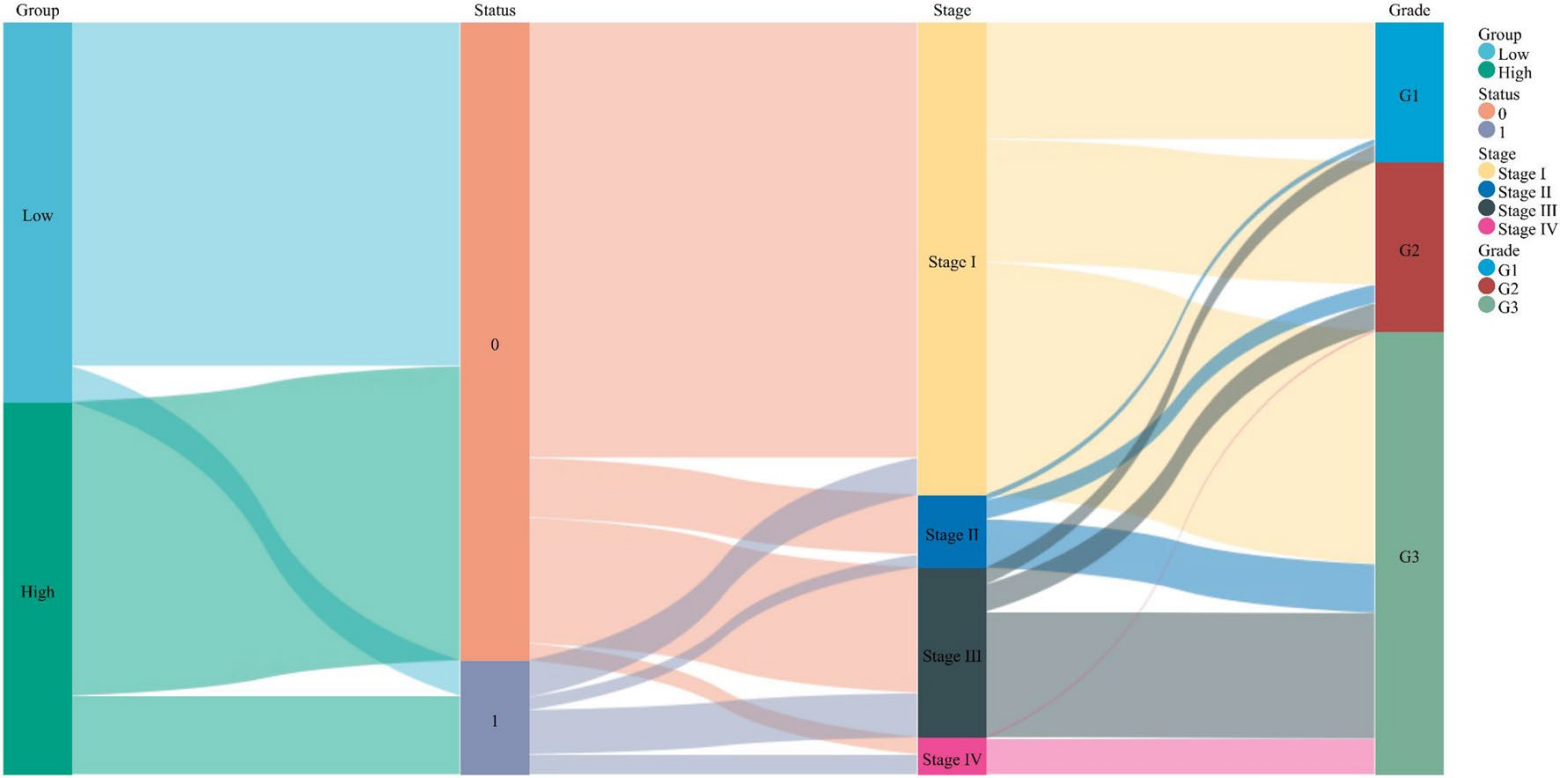
Sankey diagram of a correlation between two groups and clinical features.

**Figure 13:**
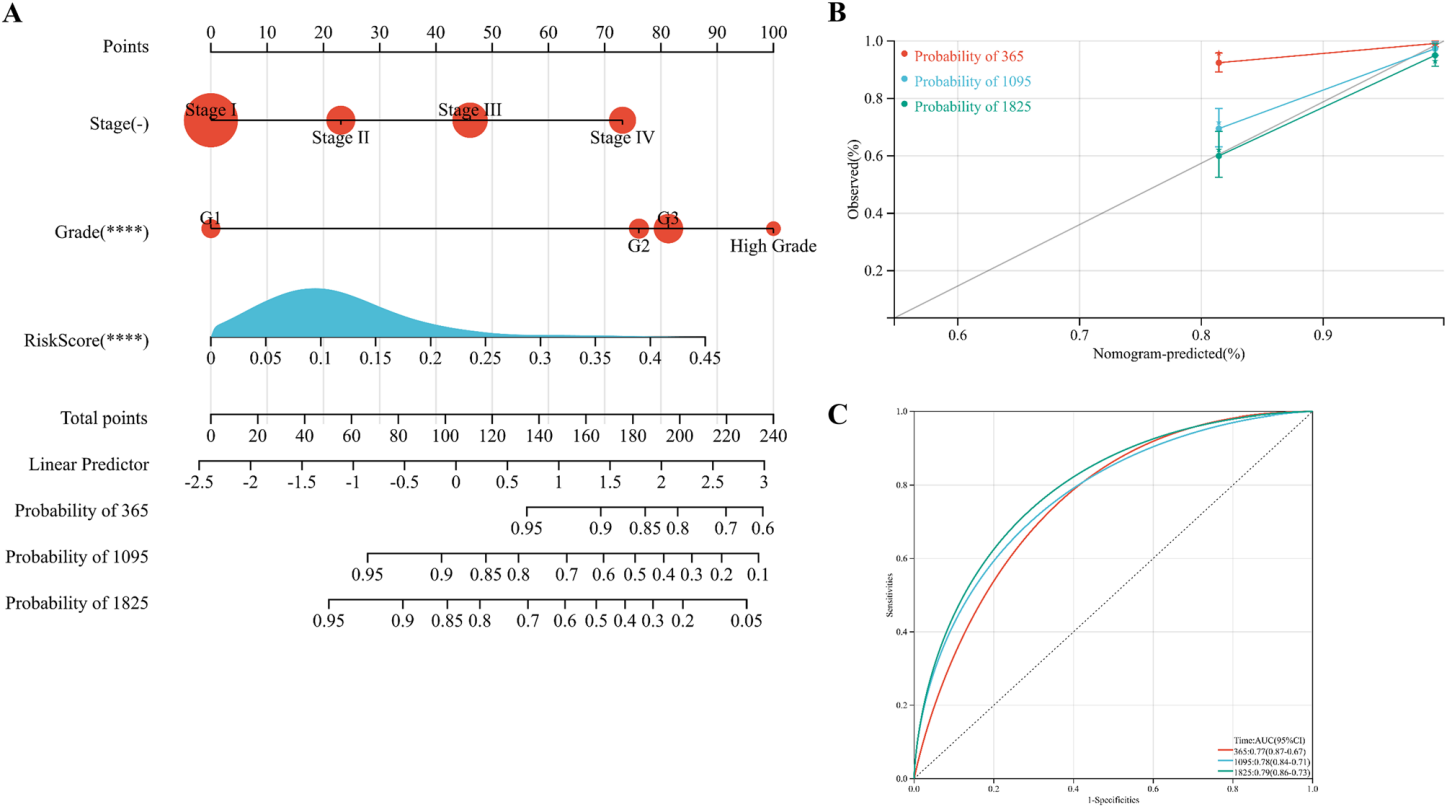
Validation of prognostic risk signature. (**A**) Nomogram integrating risk score and clinical features. (**B**) Calibration of the nomogram at 1,3, and 5 years in the training cohort. (**C**) ROC curve of the risk signature in the validation cohort.

We constructed a Sankey diagram based on the different groups and clinical features (Fig. 12). A nomogram was established to predict the survival probability of 1, 3, and 5 years (Fig. 13A). Compared with the actual 1, 3, and 5 years survival rates, the calibration curve showed that the 5 years survival rate predicted by nomogram is in good agreement with the actual survival rate (Fig. 13B).

**Figure 14:**
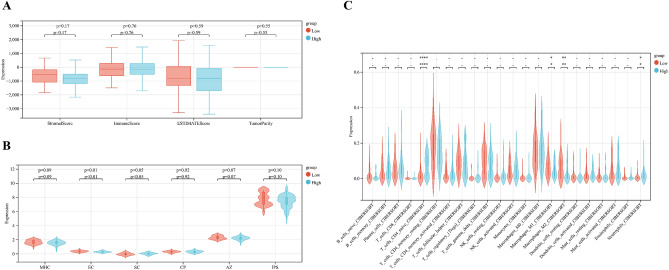
Immune cell infiltration in the training cohort. (**A**) Stromal Score, Immune Score, ESTIMATE Score, and Tumor Purity in two groups. (**B**) IPS in two groups. (**C**) Subsets of immune cell infiltration in two groups (*p < 0.05; **p < 0.01; ***p < 0.001).

We used GSE17025 to verify the stability of the prognostic model, of which AUC values of 0.77, 0.78, and 0.79 at 1, 3, and 5 years, respectively (Fig. 13C). In addition, the ESTIMATE, CIBERSORT, and IPS algorithm were performed in the validation cohort, and the results showed that the low-risk groups had higher Stromal score, higher ESTIMATE score, and higher Immune score (Fig. 14A). The IPS algorithm showed that IPS score was higher in low-risk groups than in high-risk groups (Fig. 14B), suggesting that low-risk groups are more likely to benefit from immunotherapy. The CIBERSORT algorithm indicated that naïve CD4 T cells, Macrophases-M1, and Macrophases-M2 were significantly higher in low-risk groups than in high-risk groups (Fig. 14C). These results demonstrated that the prognosis related genes may be novel biomarkers for evaluating the prognosis and TIME of UCEC.

### Experimental verification in vitro

**Figure 15:**
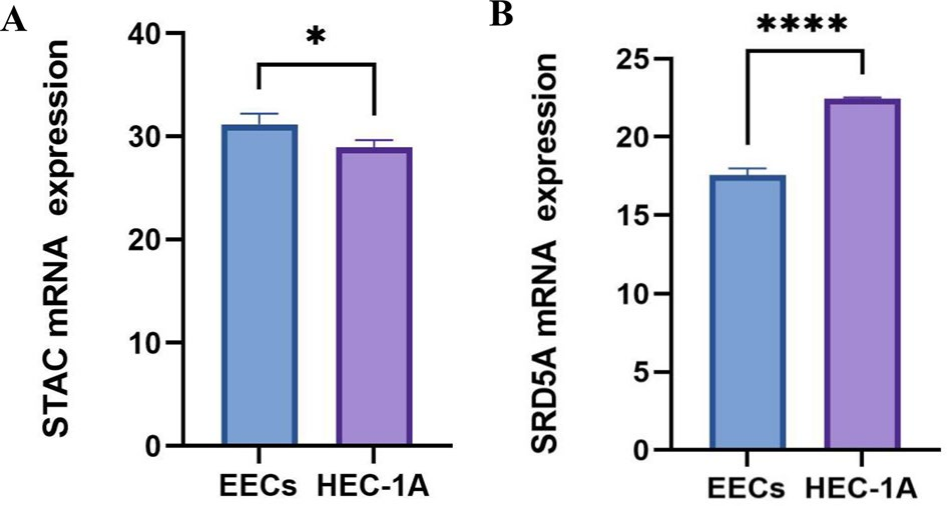
A mRNA expression level of (**A**) STAC, and (**B**) SRD5A by qRT-PCR (*p < 0.05; ****p < 0.0001).

The PCR results showed that compared with normal endometrial epithelial cells, STAC was significantly expressed lowly in HEC-1A (*p* < 0.01), and SRD5A was significantly expressed highly in HEC-1A (*p* < 0.0001) (Fig. 15A,B), indicating that STAC and SRD5A could be used as prognostic genes of UCEC.

## Discussion

UCEC is a common gynecological malignancy with an increasing incidence in recent years^28^. At present, it is considered that type I and type II non-estrogen-dependent UCEC may be related to PTEN, P16, P53, and other gene mutations. Based on the mining and analysis of the TCGA database, identifying tumor-related biomarkers and establishing a prognosis prediction model have been proven to be an effective method to predict the prognosis of tumor patients.

Firstly, we divided UCEC patients into three clusters by consistent clustering. We hypothesized that cluster C1 represents the immune inflammation type, cluster C2 represents the immune rejection type, and cluster C3 represents the immune desert type. Subsequently, we characterized TIME with CIBERSORT to compare the correlation of immune cell infiltration in three clusters, showing that cluster C1 may be more suitable for immunotherapy. DEGs in the training cohort are mainly enriched in the MAPK signaling pathway, PD-L1 expression, and PD-1 checkpoint pathway in cancer. The MAPK signaling pathway is a central pathway that regulates cellular proliferation, differentiation, and survival^29^. Although targeted therapy with MAPK pathways has produced a significant clinical response in most cancer patients, tumor recurrence rates are high due to the development of drug resistance^30^. Activation of the PD-1/PD-L1 signaling pathway can avoid peripheral tissue damage caused by excessive immune response, thereby reducing the occurrence of autoimmune diseases. However, when induced by the tumor microenvironment, the activation of PD-1 and PD-L1 as well as the PD-1/PD-L1 pathway often suppresses the T cell immune response and mediates immune escape of tumors, leading to their development^31^. Moreover, we screened out the hub genes through PPI, including BUB1, PLK1, MKI67, CDC20, KIF11, RAD51, AURKB, CENPA, AURKA, and CCNB1. The BUB1 gene plays an important role in cell division^32^. It is highly expressed in breast cancer^33^ and pancreatic cancer^34^. PLK1 regulates malignant biological behaviors such as proliferation, infiltration, and metastasis of tumor cells through interaction with tumor-related proteins such as p53, caspase 3, and golden egg white enzyme MMP-9^35^. MKI67 was proven to be associated with prognosis of the UCEC^36^. CDC20 has significant expression in both the meiosis and cell cycle sub-pathways of oocytes, and its level affects the prognosis of patients^37^. In female reproductive system tumors, KIF11 is a prognostic marker of uterine cancer and ovarian cancer^38^. The protein expression level of RAD51 in tumor tissues is significantly higher than that of normal tissues. High RAD51 expression was associated with higher tumor pathological grade, lymph node metastasis and clinical stage, and increased with increasing malignancy^39^. The expression of AURKA in normal endometrium was observed mainly in the proliferative phase. The AURKA expression was significantly increased in carcinomas compared with normal proliferative endometrium. In endometrial carcinomas, the expression of AURKB was significantly increased in high-grade tumors^40^. As an oncogenic gene, CENPA is associated with the prognosis of many cancers^41^. CCNB1 mRNA levels vary in expression in different cell cycles, with the highest expression in the G2 / M phase. CCNB1 has important prognostic value in various tumors^42^. GEPIA showed that the hub genes were all significantly expressed highly in UCEC, which is consistent with literature reports. The GSEA analysis of black module showed that the pathways enriched in the hub genes all have been proven to be related to immunity^43^.

In this study, we compared LASSO with other machine methods (elastic network and ridge regression) to further emphasize the importance of the LASSO model. LASSO is a linear regression method using L1 regularization. Using L1 regularization can make some learned feature weights zero, so as to achieve the purpose of thinning and feature selection^44^. The basic idea of LASSO is to minimize the sum of the squares of the residuals under the constraint that the sum of the absolute values of the regression coefficients is less than a constant, so that some regression coefficients strictly equal to 0 can be generated and an interpretable model can be obtained^45^. Both LASSO and ridge regression can solve the over-fitting problem to a certain extent and are more stable than the least squares method. The difference is that LASSO has the characteristics of feature selection and can obtain sparse solutions, while ridge regression can only prevent over-fitting. This is because LASSO reduces the insignificant characteristic coefficient to zero, whereas ridge regression reduces the coefficient to near zero but not zero^46^. The elastic network model is the model fusion of LASSO and ridge regression, and adopts the parallelization method. The base model is a linear model, and an LI norm and an L2 norm are added to it, which is also equivalent to adding an L2 regularization term on LASSO. However, in the process of coefficient compression, the elastic network model relaxes the screening of features^47^.

Finally, we constructed a prognostic risk signature based on prognostic genes STAC and SRD5A1. We found that the protein expressions of STAC and SRD5A1 in UCEC and normal tissues were significantly different. GEPIA showed that SRD5A1 was expressed highly in UCEC, while STAC was expressed lowly in UCEC. Patients with highly expressed STAC have short OS (*p* < 0.05). STAC and SRD5A1 both had high CDB + T cells infiltration levels, suggesting that the prognostic genes are closely related to TIME. The prognostic genes used to establish the risk signature have been shown to be closely related to tumor development. The role of STAC in tumorigenesis and progression is not clear^48^. Studies have found that STAC can promote breast cancer cell necrosis^49^. STAC was expressed highly in pancreatic cancer cells. STAC can activate SIRT1 lysosomal-dependent cell death^50^. Moreover, STAC can promote the migration of mouse tumor cells^51^. Thus, the bidirectional regulation of SATC in tumors depends on its level of activity, among other factors^52^. The SRD5A1 immunoreactivity occurs in the nucleus and cytoplasm^53^, and dihydrotestosterone (DHT) is the most effective endogenous androgen, which is converted from testosterone by SRD5A1^54^. Androgens play a role in diseases such as endometriosis^55^. SRD5A1 is immunoreactive in proliferating endometrial tissue^56^. Silencing SRD5A1 not only reduces progesterone metabolic, but also increases unmetabolized progesterone level, suggesting that SRD5A1 is a potential target for UCEC treatment^57^. The PCR results showed that compared with normal endometrial epithelial cells, STAC was significantly expressed lowly in HEC-1A (*p* < 0.01), and SRD5A was significantly expressed highly in HEC-1A (*p* < 0.0001), indicating that STAC and SRD5A could be used as prognostic genes of UCEC, and the prognostic risk model constructed by them had certain reliability. These results indicated that the prognosis genes were significantly correlated with TIME in UCEC. Thus, we speculated that the risk model we established could predict the poor prognosis of UCEC, and reflected the low immune status.

The established risk signature successfully classified the UCEC patients into high-risk and low-risk groups, the TIME and immune status of the two groups differed significantly. The nomogram could predict the prognosis of UCEC patients accurately. Besides, the low-risk groups are more likely to benefit from immunotherapy.

Although multiple studies have established relevant prognostic models in UCEC^58^, our study shows unique advantages compared with previous studies. Firstly, the number of patients was significantly different from that of the published article. Secondly, our work identified three significantly different clusters of prognosis and immune status by consensus clustering. Thirdly, genes were obtained in different ways, we have selected DEGs based on WGCNA and partially elucidated the underlying mechanisms. Fourthly, we used the GEO data set to validate the prognosis model. Fifthly, we elucidated the effects of hub genes on TIME and prognosis. Sixthly, the prognostic model we constructed is different from the previous articles. Seventhly, we performed experimental validation in vitro, and the results showed that the prognostic risk model was stable to a certain extent, which could accurately predict the prognosis of UCEC. However, the study needs more experimental verification in vivo. The establishment of prognostic risk signature provided new possibilities for us to predict the efficacy of immunotherapy, and promotes personalized treatment for UCEC patients in the future.

## Conclusion

In this study, we identified three clusters, clusters C1, C2, and C3. We speculate cluster C1 is the immune inflammation type, cluster C2 is the immune rejection type, and cluster C3 is the immune desert type. The prognosis prediction model we constructed can provide support for clinically predicting the prognosis of UCEC patients and finding the corresponding immunotherapy.

## Supplementary Information

Supplementary Table S1.

Supplementary Table S2.

## Data Availability

The datasets in this study were acquired from TCGA (https://www.cancer.gov/ccg/research/genome-sequencing/tcga/using-tcga-data/types) and GEO databases (https://www.ncbi.nlm.nih.gov/geo/query/acc.cgi?acc=GSE17025). The data used to support the findings of this study are included in the article.

## Abbreviations

UCEC: Uterine corpus endometrial carcinoma
TCGA: The cancer genome atlas
GEO: Gene expression omnibus
TIME: Tumor immune microenvironment
DEGs: Differentially expressed genes
WGCNA: Weighted correlation network analysis
HPA: Human protein atlas
PPI: Protein–protein interaction
OS: Overall survival
