# Supplementary material for: Identification of immune-related gene signature for predicting prognosis in uterine corpus endometrial carcinoma
Source: Sci Rep. 2023 Jun 7;13:9255. doi: 10.1038/s41598-023-35655-x (PMC10247783; doi:10.1038/s41598-023-35655-x)
Supplement: Supplementary file 1 — Supplementary Table S1. [file 41598_2023_35655_MOESM1_ESM.docx]

**Supplementary Table S1 Clinical features of the UCEC patient**

|  | TCGA | GEO |
| --- | --- | --- |
| No. of patients | 548 | 103 |
| Age (%) |  |  |
| ≤65 | 309(56.4) | 52(50.5) |
| ＞65 | 239(43.6) | 51(49.5) |
| Grade (%) |  |  |
| G1 | 99(18.1) | 30(29.1) |
| G2 | 122(22.3) | 36(35.0) |
| G3 | 316(57.7) | 25(24.3) |
| GX | 11(1.9) |  |
| Stage (%) |  |  |
| I | 342(62.4) | 91(88.3) |
| II | 52(9.5) |  |
| III | 124(22.6) |  |
| IV | 30(5.5) |  |
| Survival status |  |  |
| OS day（median） | 897.5 | NA |
| Ending (%) |  |  |
| Survival | 459(83.8) | NA |
| Death | 89(16.2) | NA |
