# Supplementary material for: Identification of immune-related gene signature for predicting prognosis in uterine corpus endometrial carcinoma
Source: Sci Rep. 2023 Jun 7;13:9255. doi: 10.1038/s41598-023-35655-x (PMC10247783; doi:10.1038/s41598-023-35655-x)
Supplement: Supplementary file 2 — Supplementary Table S2. [file 41598_2023_35655_MOESM2_ESM.docx]

**The primer sequences**

| **Gene** | **Sequence** **(5′-3′)** |
| --- | --- |
| SRD5A-F | GAGGCAGGAGAATCGCTTGAACC |
| SRD5A-R | CAGGCTGAGATGGTGTCTTGTGTC |
| STAC-F | CACCAGCAGCCAGGAATCCAAG |
| STAC-R  β-ACTIN-F | CAGCCTTCAGAAGCCACCTAATACC  CAGATGTGGATCAGCAAGCAGGA |
| β-ACTIN-R | CGCAACTAAGTCATAGTCCGCCTA |
